# *N*‐glycosylation and expression in human tissues of the orphan GPR61 receptor

**DOI:** 10.1002/2211-5463.12339

**Published:** 2017-11-20

**Authors:** Paweł Kozielewicz, Hatun Alomar, Syaratul Yusof, Gillian Grafton, Alison J. Cooper, S. John Curnow, James W. Ironside, Hardev Pall, Nicholas M. Barnes

**Affiliations:** ^1^ Institute of Clinical Sciences College of Medical and Dental Sciences University of Birmingham UK; ^2^ Institute of Inflammation and Ageing College of Medical and Dental Sciences University of Birmingham UK; ^3^ National CJD Research and Surveillance Unit Centre for Clinical Brain Sciences University of Edinburgh UK; ^4^ Neurology Department Queen Elizabeth Hospital Birmingham UK; ^5^ Present address: *Department of Physiology and Pharmacology Karolinska Institutet Nanna Svartz väg 2 17 177 Stockholm Sweden; ^6^ Present address: Pharmacology and Toxicology Department College of Pharmacy King Saud University Riyadh 12372 Saudi Arabia; ^7^ Present address: Faculty of Pharmacy Universiti Kebangsaan Malaysia 50300 Kuala Lumpur Malaysia

**Keywords:** GPCR, hippocampus, lymphocyte, membrane trafficking, *N*‐linked glycosylation

## Abstract

A number of members of the G protein‐coupled receptor class of cell surface receptors are ‘orphans’ with no known endogenous ligand. One of these orphan receptors is GPR61; there are little data about its expression in human cells and tissues. In this study, we investigated the post‐translational modification of GPR61 by *N‐*glycosylation at an identified consensus *N*‐glycosylation site (N12) and the impact of this modification upon the subcellular expression of the protein. The *N*‐glycosylation inhibitor tunicamycin reduced the apparent molecular weight of immunoreactivity associated with *myc*‐tagged GPR61 by 1–2 kDa, which was comparable to the evident molecular weight of the *myc*‐tagged N12S GPR61 mutant with disrupted consensus *N*‐glycosylation site. Analysis of GPR61 expression demonstrated that tunicamycin treatment reduced considerably heterologous expression of GPR61 in the cell membrane despite the N12S GPR61 mutant being readily expressed at the cell surface. These results demonstrate that GPR61 is subject to *N*‐glycosylation but suggest this is not a prerequisite for cell surface expression, although *N‐*glycosylation of other proteins may be important for cell membrane expression of GPR61. Expression of GPR61 protein was demonstrated at the cellular level in human hippocampus and human peripheral blood mononuclear cells. In the latter, there was a significantly higher expression of GPR61 in the Th17 cell subset in comparison with resting CD4+ cells, which may point toward a potential role for the GPR61 receptor in autoimmune diseases. This is the first report that GPR61 protein is subject to post‐translational modification and is expressed in immune cell subsets and the hippocampus. These findings will help guide studies to investigate the function of GPR61.

**Abbreviations**
B cells: B lymphocytes
CA 1–4: Cornu Ammonis areas of hippocampus
DG: dentate gyrus
GPCR: G protein‐coupled receptor
PBMCs: peripheral blood mononuclear cells
T cells: T lymphocytes

G protein‐coupled receptors (GPCRs) are the largest family of cell surface receptors and are the therapeutic targets for nearly a third of clinically marketed drugs 1, yet a number of human GPCRs remain poorly understood including lack of knowledge of their endogenous orthosteric ligand. Included in these so‐called orphan receptors is GPR61 (other names: GPCR3, biogenic amine receptor‐like G protein‐coupled receptor, BALGR), which is predicted to be a 49‐kDa GPCR that belongs to orphan receptors class A 2, 3.

The quaternary structure and function of this receptor have not been revealed. The initial report identifying the GPR61 clone indicated that the amino acid sequence of GPR61 shares some degree of sequence similarity (28–31%) with certain histamine, adrenergic, serotonin, and dopamine receptors 2, although it was later suggested that GPR61 might be better grouped with melatonin receptors by phylogenetic clustering 4. GPR61 mRNA is expressed across a wide range of tissues with brain, testis, and leukocytes having the highest apparent levels of expression 2, 5. Of relevance, recent immunohistochemistry (IHC) staining revealed that the zona glomerulosa has the highest expression of GPR61 within the human adrenal gland, with a potential link to aldosterone synthesis 6.

The first study reporting a potential low‐affinity inverse agonist for GPR61 was published in 2003 7; 5‐(nonyloxy)‐tryptamine (5‐NOT) impacted a chimeric receptor with high micromolar potency. Interestingly, there is some evidence for autoregulation of the receptor activity 8, its constitutive activity 9, although one previous work was not supportive 10.

Knowledge about cellular and physiological functions for GPR61 is inferred from studies with GPR61‐knockout (ko) mice. These mice display elevated levels of liver triacylglycerols, plasma leptin, and insulin 11, which may underlie the greater fat mass and correlate with lower levels of hypothalamic proopiomelanocortin and brain‐derived neurotropic factor mRNA. Such findings forward the GPR61 receptor as a target to regulate body weight and food intake and hence may provide a target for antiobesity drugs. In addition, GPR61 has also been proposed to play a role in aldosterone production 12.

Given the potential of the GPR61 receptor as a drug target for therapeutic benefit, we have studied the potential importance of *N*‐glycosylation of GPR61 protein – the functions of many GPCRs are influenced by this post‐translational modification 13, 14 – and also investigated the presence of the protein in some human tissues.

## Materials and methods

### Cell culture and cloning

**Figure 1:**
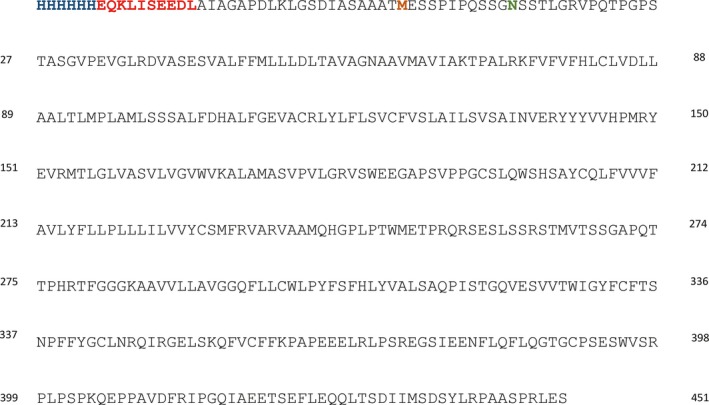
Amino acid sequence of the mature human recombinant GPR61 protein, highlighting the His‐tag (in blue), *myc*‐tag (in red), the beginning of the native sequence (methionine in orange), and the potential *N*‐glycosylation site (in green).

Wild‐type GPR61 plasmid DNA (R&D Systems, Minneapolis, MN, USA) was inserted into pCMV6‐An‐His‐Myc vector (OriGene, Rockville, MD, USA) using *Hin*dIII and *Xho*I restriction enzymes (Fig. 1). The N12S mutant was generated using Q5 site‐directed mutagenesis kit (NEB, Ipswich, MA, USA) by PCR DNA amplification with the following primers: 5′‐TCATCAGGGAGCTCTTCCACTTTG‐3′ and 5′‐CTGGGGGATGGGTGAGGA‐3′ according to the manufacturer's instructions. Plasmid cDNA was then transformed into XL10 gold‐competent cells (Stratagene, San Diego, CA, USA) and isolated using Plasmid Maxi Kit (Qiagen, Hilden, Germany). The identity of DNA was confirmed by DNA sequencing. The plasmid concentration was determined using NanoDrop 2000 (Thermo Fisher Scientific, Waltham, MA, USA).

Plasmid cDNA (10 μg) was transfected into ~ 90% confluent HEK293 cells of early passage (< 20, cells coming from our laboratory's repository) using Lipofectamine 2000 reagent (Thermo Fisher Scientific), and stably transfected cells were selected with geneticin (500 μg·mL^−1^; Thermo Fisher Scientific) over a period of 4–5 weeks. The GPR61‐expressing cells were maintained in Dulbecco's modified Eagles’ medium, supplemented with 10% (v/v) fetal bovine serum, 1% (v/v) penicillin/streptomycin (10 000 U·mL^−1^ penicillin and 10 mg·mL^−1^ streptomycin), and geneticin (250 μg·mL^−1^). The cells were maintained at 37 °C, 5% CO_2_, 95% relative humidity.

### Antibodies to GPR61

Specificity of commercially available anti‐GPR61 antibody (sc‐54599, SCT) was tested in flow cytometry and western blot using recombinant HEK293 cells, and it was confirmed that this antibody cannot be used to effectively assay for GPR61 expression in these cells. GPR61 antisera were provided by A. Davenport, and it was shown that GPR61‐transfected cells have significantly higher staining levels in flow cytometry than untransfected cells.

### SDS/PAGE and western blotting

Cells were scraped off the culture flask and sonicated over wet ice. Following the sonication, samples were resuspended in an equal volume of 2× radioimmunoprecipitation assay buffer (RIPA; Cell Signaling Technology, Danvers, MA, USA) and appropriate volume of 2× urea sample buffer. No more than 30 μg of protein was loaded and run on 4–20% Tris/glycine gels (Bio‐Rad, Hercules, CA, USA, cat. 4561094). For each gel, at least one molecular weight ladder was included (New England Biolabs, Ipswich, MA, USA, cat. P7712). Proteins were transferred onto PVDF membranes, which were then blocked in 10% nonfat milk (Marvel) for 2 h at room temperature. Membranes were incubated in 1 : 3000 anti‐*myc* (Cell Signaling Technology) or 1 : 3000 anti‐GAPDH (Abcam, Cambridge, UK) in 5% nonfat milk overnight at 4 °C before washing at room temperature. Membranes were incubated subsequently in anti‐mouse or anti‐rabbit HRP‐linked secondary antibody (Cell Signaling Technology) in 5% nonfat milk in wash buffer at room temperature for 2 h. Membranes were washed and then incubated in ECL reagent for 5 min. The membrane was subsequently exposed to film and developed. Alternatively, the membrane was developed using ChemiDoc MP system imager (Bio‐Rad) that also allows for the subsequent quantification of chemiluminescent bands. Images acquired using ChemiDoc MP were analyzed in image lab 5.2.1 software (Bio‐Rad).

### Immunocytochemistry

Cells were seeded at a density of 50 000 cells on poly‐d‐lysine‐coated coverslips and cultured for 18 h at 37 °C. The cells were then washed in PBS and fixed with 2% formaldehyde solution (Sigma Aldrich, St Louis, MO, USA, cat. F8775) for 15 min at room temperature and protected from light. After washing, for staining intracellular proteins, cells were permeabilized with 0.3% Triton X‐100 (Sigma Aldrich, cat. X100) in PBS for 20 min at room temperature. The cells were then washed with PBS and blocked in 10% FBS in PBS (blocking buffer) for 1 h. After the blocking step, primary antibody solution (1 : 3000 anti‐*myc* or 1 : 2000 GPR61 antisera) in blocking buffer was added and incubated overnight at 4 °C. On the following day, primary antibody was removed by washing with PBS. Secondary antibody solution (1 : 1000 fluorochrome‐conjugated secondary antibody; Thermo Fisher Scientific) in blocking buffer was then added and incubated for 3 h at room temperature. Cells were then washed in PBS. In the experiments in which *myc‐*tagged GPR61 was used to stain extracellularly and intracellularly, cells were permeabilized on the second day after the first application of the secondary antibody. The procedure was then repeated: Cells were incubated overnight at 4 °C in a primary antibody solution. On the following day, the cells were washed and a different secondary antibody conjugated to a different fluorochrome at the same concentration was added in blocking buffer and left to incubate for 3 h at room temperature. After the incubation with the secondary antibody solution, the cells were washed with PBS. The coverslips containing the cells were then mounted on microscope slides with VectaShield Hardset mounting medium containing DAPI (Vector Laboratories, Burlingame, CA, USA, cat. H‐1500). Images were captured on the same day using Zeiss LSM Meta 510 confocal microscope and analyzed in lsm image browser software (Zeiss, Oberkochen, Germany).

### *Myc* immunoreactivity by flow cytometry

HEK293 cells stably expressing His‐*myc‐*tagged GPR61 and untransfected HEK293 cells were used. Some cells were treated with 1.0 μg·mL^−1^ tunicamycin (Sigma Aldrich) for 48 h to inhibit the *N*‐glycosylation. Following the treatment, medium was aspirated from the cells and the cells were washed in PBS. The cells were then detached from the flask by the addition of 1 mm EDTA PBS and were left at 4 °C for 10 min. The cells were transferred to complete DMEM and centrifuged twice at 400 ***g*** for 5 min. The supernatant was discarded and the cells were resuspended in 0.5% BSA/PBS. The cells were resuspended in 1 : 500 mouse anti‐*myc* antibody in 0.5% BSA/PBS and incubated for 1 h at 4 °C. The cells were then washed twice by centrifugation and resuspended in 1 : 200 goat anti‐mouse PE (Abcam) in 0.5% BSA in PBS. The cells were incubated for 30 min at room temperature. The cells were then washed twice by centrifugation before resuspension in 0.5% BSA/PBS and immediately assayed using ADP Cyan flow cytometer (Beckman Coulter, Brea, CA, USA).

### Hippocampus tissue samples and immunohistochemistry

Human formalin‐fixed, paraffin‐embedded hippocampal samples were obtained from the Medical Research Council, Edinburgh Brain and Tissue Bank (University of Edinburgh, Edinburgh, UK). The donors (aged 20–40, postmortem index 36–77 h) were free from neurological or psychological conditions. The study was approved by the University of Birmingham Ethics Committee.

The immunohistochemistry staining was conducted as previously described by Brady *et al*. 15.

### Isolation of human peripheral blood mononuclear cells

Peripheral blood mononuclear cells (PBMCs) were isolated from whole blood from healthy donors or from leukocyte reduced system (LRS) cones using Ficoll–Paque (GE Healthcare, Little Chalfont, UK). In some experiments, cells were stimulated in the presence of PHA‐L (5 μg·mL^−1^; Roche, Basel, Switzerland) for 24 h.

### RNA isolation from human hippocampus and PBMCs

Total RNA was isolated from human PBMCs using QIAGEN RNeasy Mini Kit according to the manufacturer's instructions with DNase treatment included. RNA concentration and purity were measured on NanoDrop 2000, and RNA was stored at −80 °C prior to the experiments.

### Real‐time PCR analysis of GPR61 expression in human PBMCs

The Precision OneStep qRT‐PCR mastermix, β‐actin, and GPR61 primers were obtained from PrimerDesign. The GPR61 primer sequences were as follows: 5′‐CTTTCGAATCCCAGGCCAGA‐3′ (forward primer) and 5′‐GCAGGACGGAGGTAGCTG‐3′ (reverse primer).

### GPR61 expression by PBMCs assessed by flow cytometry

PBMCs were stained with a panel of antibodies specific for cell surface markers (mouse anti‐human CD4, CD8, CD14, and CD19; eBioscience, San Diego, CA, USA) for 20 min at 4 °C in 10% FcR blocker (Miltenyi, Bergish Gladbach, Germany). The cells were then washed twice, fixed in Cytofix/Cytoperm (BD) for 15 min at 4 °C, and permeabilized by washing in Perm buffer (Becton Dickinson, Franklin Lakes, NJ, USA) before being stained with GPR61 antisera or control preimmune sera for 30 min at 4 °C. The cells were then washed twice and stained with AlexaFluor647‐conjugated anti‐goat antibody (1 : 2000; Thermo Fisher Scientific). The cells were washed twice and assayed on ADP Cyan flow cytometer.

For the assessment of GPR61 expression in IFN‐γ+ and IL‐17+ cells, PBMCs were stimulated for 24 h with 5 μg·mL^−1^ phytohemagglutinin‐M at 37 °C. After 24 h, the plate was centrifuged and the cells were restimulated with 50 ng·mL^−1^ phorbol 12‐myristate‐13‐acetate and 750 ng·mL^−1^ ionomycin in the presence of 0.5 μL per well GolgiStop for 3 h at 37 °C. After 3 h, the cells were washed twice by centrifugation. Cells were stained for surface markers as above. Cells were then fixed and incubated for 15 min at 4 °C in the dark. The cells were then centrifuged twice in 1× Perm/Wash buffer. The cells were stained with anti‐GPR61, anti‐IFN‐γ, and anti‐IL‐17 antibodies for 20 min at 4 °C. The cells were centrifuged twice and stained with the AlexaFluor647‐conjugated anti‐goat antibody. The cells were washed twice and assayed on ADP Cyan flow cytometer.

### Statistical analysis

Statistical analysis was performed using prism 6 (GraphPad, La Jolla, CA, USA).

## Results

### SDS/PAGE/western blotting

**Figure 2:**
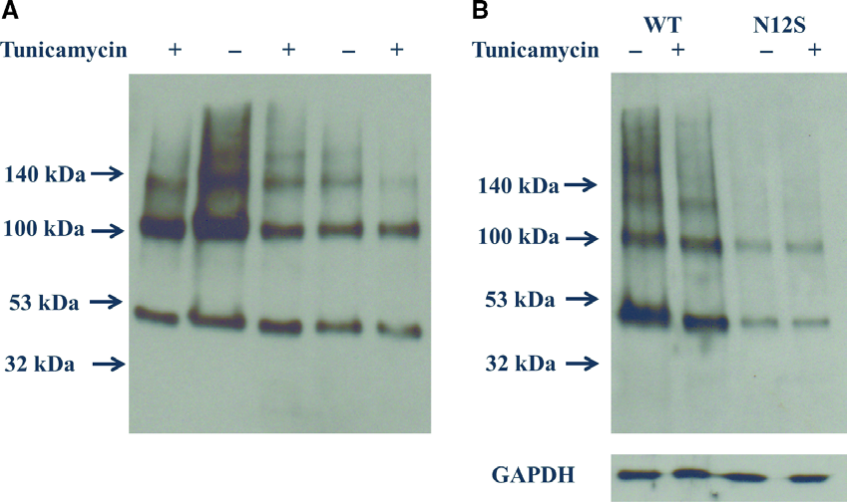
(A) Prevention of *N*‐glycosylation of GPR61 by tunicamycin reduces the molecular weight of the predominant *myc*‐immunoreactive species arising from stable expression of the nonmutated myc‐tagged GPR61. (B) The consensus *N*‐glycosylation sequence at N12 was disrupted by mutagenesis (N12S) with the arising *myc*‐immunoreactive species displaying a lower apparent molecular weight compared with the nonmutated protein, and furthermore, the N12S mutant apparent molecular weight was unaffected by tunicamycin treatment. Data presented are from a single representative experiment, which was repeated at least five times with similar results. GAPDH was used as a loading control.

SDS/PAGE separation of lysates of HEK293 stably expressing nonmutated and N12S mutant *myc*‐tagged GPR61 identified bands of immunoreactivity consistent with the size of the predicted molecular weight of the *myc*‐tagged GPR61 receptor ~ 53 kDa (Fig. 2). The stably transfected cells also gave rise to a secondary band at about 110 kDa, which may suggest the formation of a dimer. Addition of tunicamycin (1.0 μg·mL^−1^) to the cell culture medium for 48 h reduced consistently the apparent molecular weight of resulting *myc‐*tagged GPR61 immunoreactivity by 1–2 kDa (Fig. 2), which was not apparent for the control protein GAPDH. This was further investigated by the mutation of the predicted *N*‐glycosylation site in the N terminus of the *myc*‐tagged GPR61 (N12), which also resulted in the arising protein having a reduced molecular weight of the predominant immunoreactive species by 1–2 kDa compared with the nonmutated protein. Tunicamycin‐treated *myc‐*tagged GPR61 and *myc‐*tagged N12S cell lysates showed the same *myc* immunoreactivity pattern. These results indicate that the *myc‐*tagged GPR61 is *N*‐glycosylated.

### Immunocytochemistry

**Figure 3:**
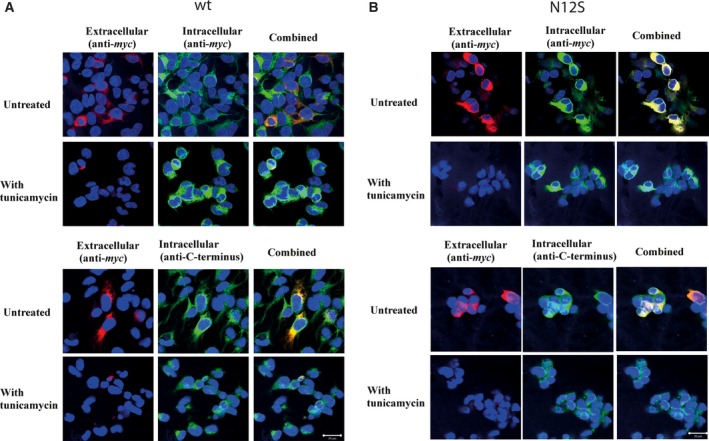
Effect of tunicamycin treatment (48 h) on the cellular localization of GPR61 immunoreactivity in HEK293 stably transfected with nonmutated *myc*‐GPR61. (A) Upper panel: Immunocytochemical detection of *myc* immunoreactivity allowed detection of the nonmutated GPR61 in unpermeabilized cells indicating protein expression in the cell membrane (red immunofluorescence). Subsequent permeabilization of cells followed by re‐immunolabeling allowed visualization of *myc*‐immunoreactive protein expressed throughout the cell (green immunofluorescence). DAPI stain (blue fluorescence) was used to visualize the cell nuclei. When the cell images were combined, immunoreactive protein expressed in the cell membrane appears yellow, whereas intracellular immunoreactive protein appears green. Treatment of cells with tunicamycin (1.0 μg·mL^−1^, 48 h) resulted in the reduced level of cell membrane *myc* immunoreactivity shown as reduced levels of red staining. Lower panel: The same as in the upper panel but the intracellular staining following the permeabilization was performed using anti‐GPR61 sera and the resulting immunoreactivity appears green. The scale bar = 20 μm. Cells are representative within a population with independent experiments repeated four times. (B) Upper panel: Immunocytochemical detection of *myc* immunoreactivity allowed detection of the N12S GPR61 in unpermeabilized cells indicating protein expression in the cell membrane (red immunofluorescence). Subsequent permeabilization of cells followed by re‐immunolabeling allowed visualization of *myc*‐immunoreactive protein expressed throughout the cell (green immunofluorescence). DAPI stain (blue fluorescence) was used to visualize the cell nuclei. When the cell images were combined, immunoreactive protein expressed in the cell membrane appears yellow, whereas intracellular immunoreactive protein appears green. Treatment of the cells with tunicamycin (1.0 μg·mL^−1^, 48 h) resulted in the reduced level of cell membrane *myc* immunoreactivity shown as reduced levels of red staining. Lower panel: The same as in the upper panel but the intracellular staining following the permeabilization was performed using anti‐GPR61 sera, and the resulting immunoreactivity appears green. The scale bar = 20 μm. Cells are representative within a population with independent experiments repeated four times.

Immunocytochemical studies on the transfected fixed HEK293 performed with either anti‐*myc* or anti‐GPR61 sera confirmed that *myc‐*tagged GPR61 is expressed both at the surface and inside the cells (Fig. 3A,B). Cells treated with tunicamycin displayed considerably reduced levels of *myc‐*tagged GPR61 immunoreactivity when the cells were stained with anti‐*myc* antibody before permeabilization with no apparent difference in the intracellular staining between treated and untreated cells. When the *N*‐glycosylation site of *myc‐*tagged GPR61 was disrupted (N12S), the staining corresponding to extracellular epitope in the mutant was similar to that of the nonmutated protein. However, following the tunicamycin treatment, the *myc‐*tagged N12S also showed markedly reduced levels of surface *myc* staining, when compared with untreated cells. These results point toward the lack of a direct role for *N‐*glycosylation of the *myc‐*tagged GPR61 protein in the cell membrane trafficking of this protein.

Under the same conditions, no immunofluorescence was detected when preimmune sera replaced the primary sera (data not shown). The distribution of intracellular staining revealed by either *myc* antibody or the anti‐GPR61 antibody was near identical, indicating that these antibodies target the same protein.

### Flow cytometry

**Figure 4:**
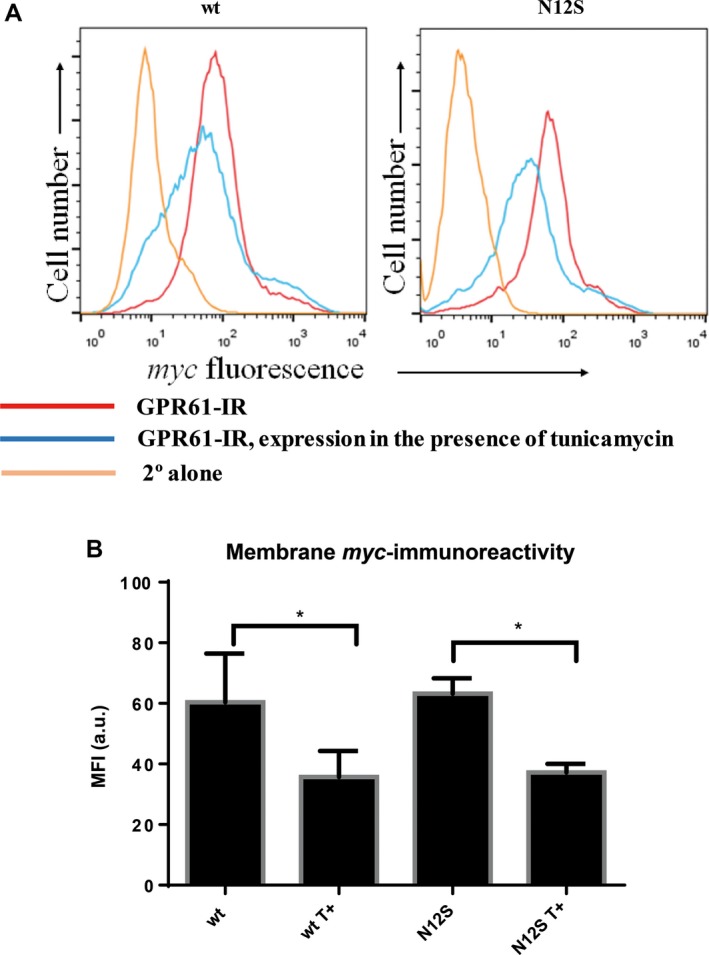
Tunicamycin (T+) reduces membrane expression of nonmutated and N12S mutant *myc*‐tagged GPR61 in stably transfected HEK293 cells. (A) Flow cytometry plots (representative from five independent experiments) showing the reduced cell surface expression of *myc*‐tagged GPR61 in nonmutated (left plot) and N12S mutant (right plot)‐transfected cells. *Myc* immunoreactivity of the cells untreated with tunicamycin is represented in red. Treatment with tunicamycin (blue) causes a decrease in the membrane *myc* immunoreactivity, indicating the lower levels of cell membrane expression of GPR61. The nonspecific immunofluorescence was determined by staining with the secondary antibody only, under the same conditions (B) Median fluorescence intensity (in arbitrary units, a.u.) of extracellular myc immunoreactivity shown as mean MFI ± SEM (*n* = 5). Tunicamycin reduces the level of membrane GPR61 expression by ~ 40% in nonmutated and N12S mutant GPR61‐stably expressing cells; *P < 0.05 (paired t‐test, analyzed for wt vs. wt T+, and N12S vs. N12S T+).

Flow cytometric analysis of expression of *myc‐*tagged GPR61 and N12S in the transfected HEK293 cells gave comparable results to immunocytochemistry (Fig. 4A) but allowed better quantification of the immunoreactivity. As opposed to the immunocytochemical method, nonfixed cells were used in order to minimize the risk of cell permeabilization and prevent any intracellular staining. Using flow cytometry, we have demonstrated that both nonmutated and N12S forms of GPR61 were transported to the cell membrane and their cell membrane expression was reduced by approximately 40% following tunicamycin treatment (Fig. 4B).

### Expression of GPR61 in human hippocampus

**Figure 5:**
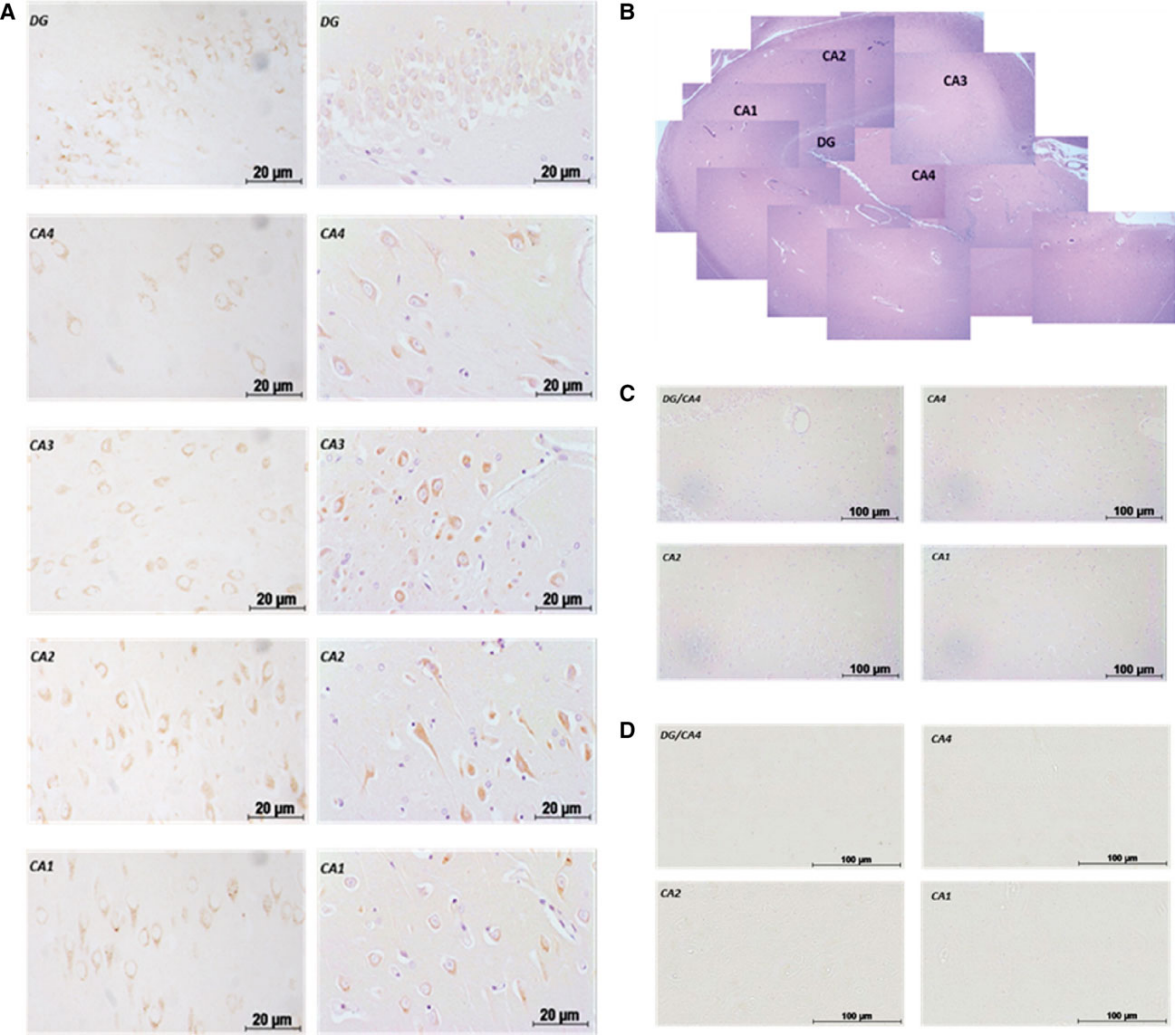
GPR61 protein expression in human hippocampal subfields (*n* = 3, representative result of five different experiments). (A) GPR61 protein immunoreactivity with posthematoxylin counterstain. (B) Histological staining and the localization of the selected immunoreactivity signals. (C) No primary antibody. (D) GPR61 preimmune sera. Scale bar = 20 μm, 100 μm.

GPR61 immunoreactivity was detected at different levels across hippocampal subfields (Fig. 5A–C). The GPR61 protein was relatively highly expressed in cells with the morphology of pyramidal neurons within subfields CA1, CA2, and CA3 of the hippocampus, and at lower levels in the granule cells of the dentate gyrus (DG) and large neurons in CA4 subfield with the morphology of GABAergic interneurons (Fig. 5A). Immunoreactivity was not apparent when preimmune sera exchanged the anti‐GPR61 sera.

### Expression of GPR61 in immune cells

**Figure 6:**
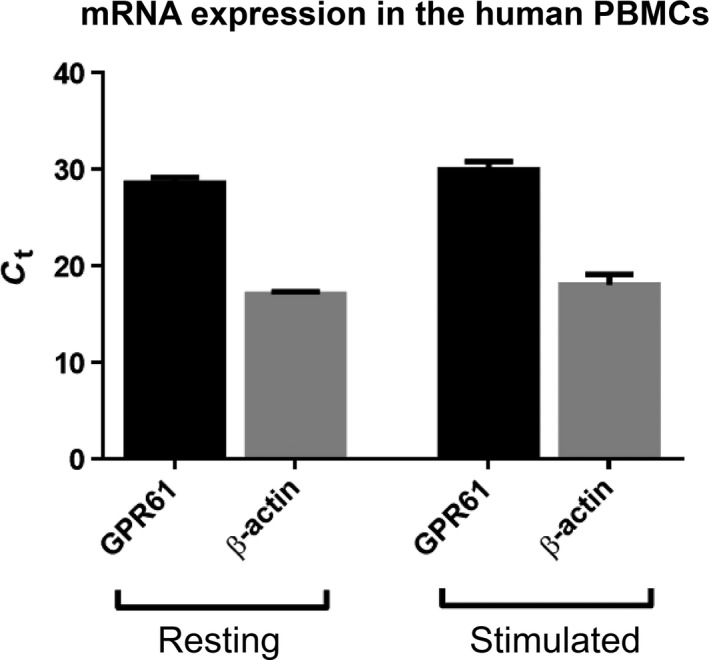
mRNA GPC61 expression in the resting and stimulated (PHA 5 μg·mL^−1^, 24 h) human PBMCs. β‐Actin was used as housekeeping gene. Data represented as mean ± SEM from three independent experiments.

**Figure 7:**
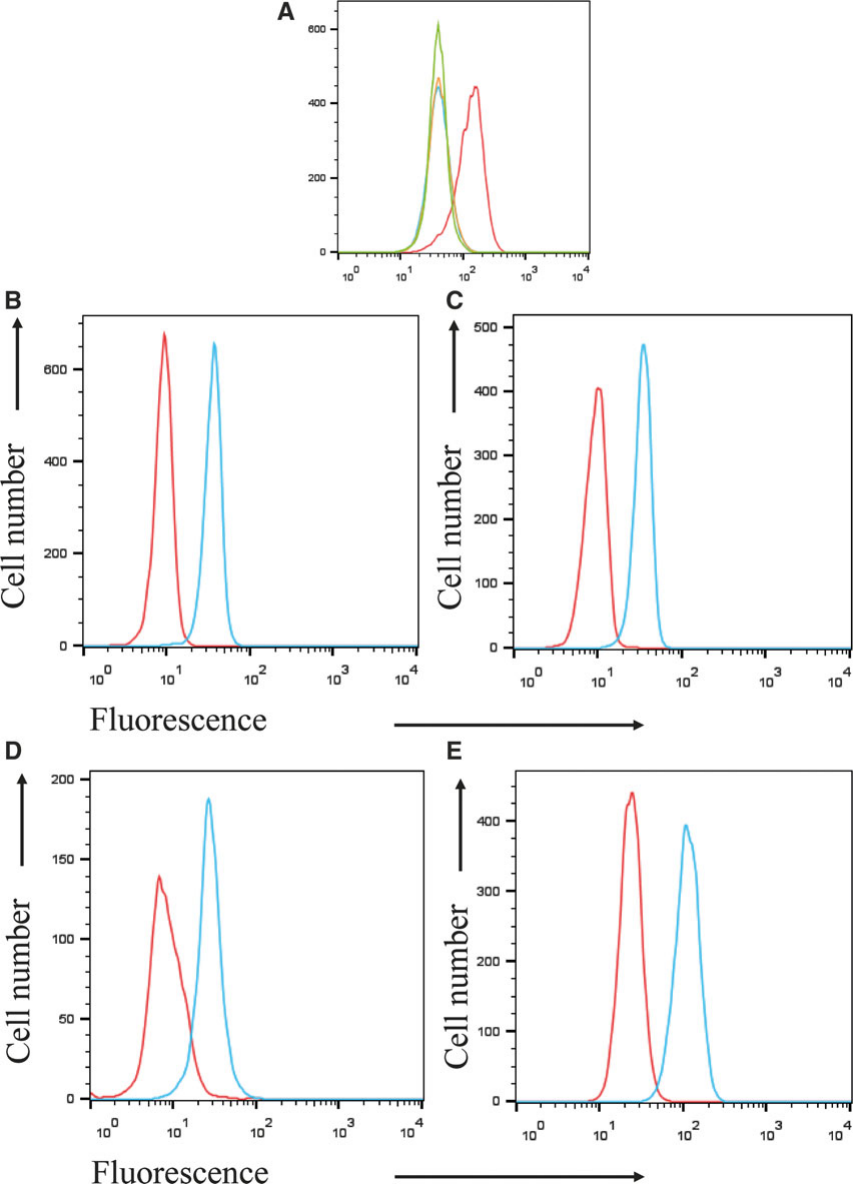
GPR61 protein immunoreactivity in human PBMCs. (A) Specificity of GPR61 antisera was tested on HEK293 stably expressing *myc*‐GPR61. Blue – untransfected HEK293 cells stained with preimmune sera; orange – untransfected HEK293 stained with GPR61 antisera; green – HEK293 cells expressing *myc‐*GPR61 stained with preimmune sera; red – HEK293 cells stably expressing *myc*‐GPR61 stained with GPR61 antisera. (B) CD4+ T cells, (C) CD8+ T cells, (D) CD19+ B cells, (E) CD14+ monocytes. PBMCs were isolated from healthy donors and stained with cell surface markers specific for each subset. Cells were then permeabilized and the GPR61 immunoreactivity was assessed by staining with GPR61 antisera (blue) or preimmune control serum (red). The figure shows a representative example from three independent experiments.

GPR61 mRNA expression was evident in human PBMCs with no real change in expression following activation of the cells with PHA (Δ*C*_t_ = 28.52 and 29.93, respectively, Fig. 6). GPR61 expression was quantified by flow cytometry and was evident in all the investigated cell subsets at similar levels (Fig. 7B–E).

**Figure 8:**
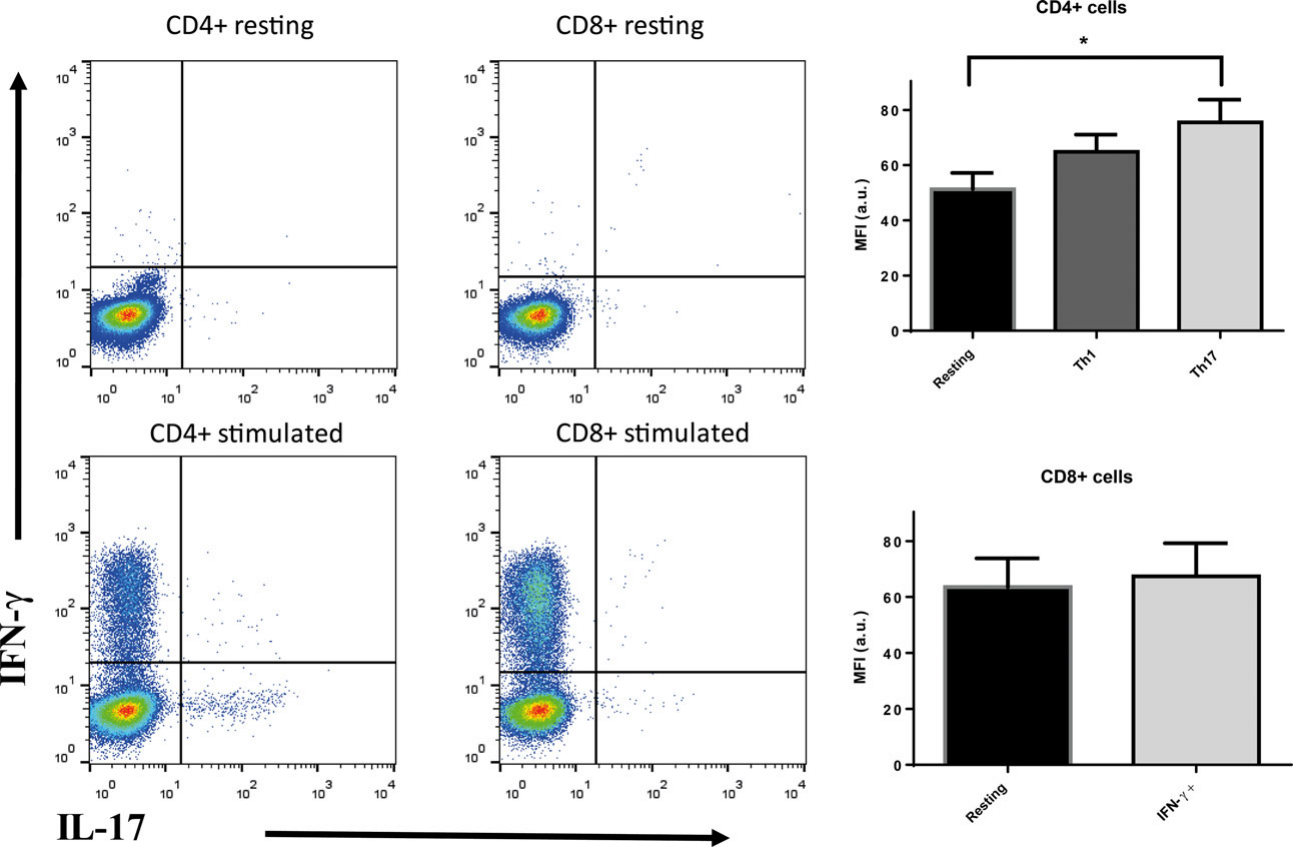
Left: representative flow cytometry plots showing the gating strategy for IFN‐γ and IL‐17 staining in PHA‐M/PMA:ionomycin activated CD4+ and CD8+. Right: median fluorescence intensity (MFI in arbitrary units, a.u.) of GPR61 immunoreactivity represented as mean ± SEM, *n* = 6; *P < 0.05, one‐way ANOVA with Dunnett's post‐test. The differences in preimmune sera immunofluorescence in these cell subsets were not significantly different (not shown).

In this study, we measured the expression of GPR61 receptor in CD4+ Th1 and Th17 cells and IFN‐γ‐producing CD8+ cells. Specificity of the GPR61 antisera was tested using recombinant HEK293 cells (Fig. 7A). Following the intracellular staining, GPR61 receptor expression was apparent in resting CD4+ and CD8+, stimulated Th1 CD4+, effector CD8+ producing IFN‐γ and CD4+ Th17 cells with CD4+ Th17 expressing significantly higher levels of GPR61 than resting and Th1 cells (Fig. 8).

## Discussion

GPR61 is an orphan GPCR with little information concerning a role and very few published reports of protein expression patterns. GPR61 mRNA expression is relatively high in brain, leukocytes, and testis 2.

In this work, we have assessed the impact of *N*‐glycosylation on the cellular expression of this GPCR in the stably transfected HEK293 cells. *N‐*glycosylation has been shown to have a major role in cell trafficking and protein folding 16. By the use of the *N*‐glycosylation inhibitor, tunicamycin, and also the generation of a non‐*N*‐glycosylated mutant, N12S GPR61, it was evident that GPR61, when expressed in human cells, is prone to *N*‐glycosylation. However, our results also indicate that while *N*‐glycosylation of GPR61 is not required for insertion into the cell membrane, *per se*, nonselective inhibition of all *N*‐glycosylation does prevent GPR61 insertion into the cell membrane, suggesting that *N*‐glycosylated ‘chaperone’ proteins support GR61 membrane trafficking, which become nonfunctional when *N*‐glycosylation is inhibited. Given the difficulty in identifying a functional activity for GPR61, it would be tempting to speculate that the receptor may function as a heterodimer and the delivery of the heteromeric dimer to the cell membrane was dependent on *N*‐glycosylation of GPR61's partner GPCR. One of the potential candidates has been forwarder in the most recent paper in which it was suggested that GPR61 and also other members of its cluster, GPR62 and GPR135, may form a heterodimer with melatonin MT2 receptor upon overexpressing both receptors 9.

The fact that GPR61 is subject to *N*‐glycosylation shows that it undergoes the same processes as many other GPCRs 14. The impact of *N*‐glycosylation on a membrane expression and a structure of ligand‐binding domain has been shown for several GPCRs, with the examples including 5‐HT_5A_ 17, β2‐adrenergic 18, AT1 angiotensin 14, or B2‐bradykinin 19.

There are a number of GPCR‐interacting proteins that, for example, influence the surface trafficking and receptor signaling, and belong predominantly to heat shock protein (Hsp), RAMP, and PDZ‐domain families. Some of these interacting proteins (e.g., RAMP2) are subject to *N*‐glycosylation 20. Presumably, other components of the machinery that are active in GPR61 cell membrane trafficking can also be impacted.

In order to be able to predict potential cellular functions of GPR61, we have investigated expression in human native tissues and cells with cellular resolution; previous reports of GPR61 expression merely reported expression by PCR, which showed relatively high levels of GPR61 mRNA in human brain, leukocytes, and testis 2. Similarly, GPR61 mRNA expression was found to be at relatively high or moderate levels in mouse CNS (hypothalamus, olfactory bulb, striatum, and hippocampus) and testis 21.

In this study, we have used two antibodies – a goat polyclonal antibody that recognizes the C terminus (H‐Cys‐Ser‐Asp‐Ser‐Tyr‐Leu‐Arg‐Pro‐Ala‐Ala‐Ser‐Pro‐ Arg‐Leu‐Glu‐Ser‐OH) of GPR61 protein and a mouse monoclonal antibody recognizing *myc‐*tag added to the N terminus of the GPR61 receptor – to study the expression of native and *myc‐*tagged GPR61 protein, respectively. In this study, we show that these two antibodies work effectively in immunocytochemistry and flow cytometry assays. Immunocytochemical staining of *myc‐*tagged GPR61‐transfected HEK293 cells demonstrated that these two antibodies indeed target the same protein. Additionally, the anti‐*myc* antibody was used in western blot studies where the polyclonal goat antibody targeting the native protein failed to give satisfactory results.

GPR61 protein expression was evident in the human hippocampus; hippocampal GPR61 immunoreactivity was widely expressed in the hippocampus at differing levels. Strong immunoreactivity signals were evident in the CA1, CA2, and CA3 subfields of the hippocampus associated with cells often displaying the morphology of pyramidal neurons. More moderate expression was detected in the DG and CA4 subfields. Hippocampal GPR61 expression in the hippocampus, particularly when associated with pyramidal neurons, suggests an involvement of this receptor in higher brain functions, such as learning, memory, and cognition 22, 23.

Our study also revealed that human PBMCs express GPR61 protein (and also confirming the expression of mRNA from our studies and reports in the literature; 2). We subsequently investigated subsets of human PBMCs using flow cytometry and demonstrated expression in the CD4+, CD8+, CD14+, and CD19+ cell subsets isolated from blood from healthy volunteers, although these subsets appeared to express similar levels of the receptor. Examples of other relatively uniformly expressed membrane receptors in leukocytes include β‐adrenergic and serotoninergic receptors 24. GPCRs can play a major role in functioning and the development of immune responses, and many GPCRs have been reported to take part in transcriptional regulation 25.

Perhaps more interestingly, within the CD4+ subset, expression of GPR61 was higher in Th17 cells compared to Th1 cells and significantly higher than in resting CD4+ T cells. Th17 cells have been intensively studied in recent years since their discovery in early 2000s. These cells are a lineage of CD4+ T cell that produces IL‐17 and appears to have a pathophysiological role in certain inflammatory diseases (e.g., multiple sclerosis, rheumatoid arthritis, psoriasis). It has been demonstrated that a shift from Th1 to Th17 phenotype may be modified by GPCRs 26. On the other hand, antagonizing another GPCR – EP4 receptor for E2 prostaglandin – inhibited Th17 expansion and was reported as effective in contact hypersensitivity, EAE, and arthritis models 27, 28. Given the lack of reports about physiological effects mediated by GPR61, a potential role in Th17‐dependent immunity and autoimmunity may warrant further investigation.

To conclude, we are reporting for the first time that GPR61 protein is subject to *N*‐glycosylation, which is consistent with many other GPCRs. In addition, this membrane GPCR is expressed in the human brain (hippocampus) and in T cells, B cells, and monocytes with higher levels of protein expression in IL17‐producing CD4+ T cells; such expression may help in the search for cellular functions mediated by this receptor, which may forward the receptor as a potential therapeutic target.

## Author contributions

GG, AJC, SJC, JWI, HP, and NMB supervised the study; PK, HA, SY, GG, and NMB designed experiments and analyzed data; PK, HA, and SY performed experiments; PK, HA, GG, SJC, and NMB wrote the manuscript; all authors approved the final version of the manuscript.
